# Preparation of Gelatin and Gelatin/Hyaluronic Acid Cryogel Scaffolds for the 3D Culture of Mesothelial Cells and Mesothelium Tissue Regeneration

**DOI:** 10.3390/ijms20184527

**Published:** 2019-09-12

**Authors:** Hao-Hsi Kao, Chang-Yi Kuo, Kuo-Su Chen, Jyh-Ping Chen

**Affiliations:** 1 Division of Nephrology, Chang Gung Memorial Hospital, Keelung 20401, Taiwan; luhuichun@adm.cgmh.org.tw (H.-H.K.); cksdavid@cgmh.org.tw (K.-S.C.); 2 Department of Chemical and Materials Engineering, Chang Gung University, Kwei-San, Taoyuan 33302, Taiwan; onesky1997@gmail.com; 3 School of Medicine, Chang Gung University, Kwei-San, Taoyuan 33303, Taiwan; 4 Department of Plastic and Reconstructive Surgery and Craniofacial Research Center, Chang Gung Memorial Hospital, Linkou, Kwei-San, Taoyuan 33305, Taiwan; 5 Research Center for Food and Cosmetic Safety and Research Center for Chinese Herbal Medicine, College of Human Ecology, Chang Gung University of Science and Technology, Taoyuan 33302, Taiwan; 6 Department of Materials Engineering, Ming Chi University of Technology, Tai-Shan, New Taipei City 24301, Taiwan

**Keywords:** cryogel, scaffold, mesothelial cells, gelatin, hyaluronic acid, 3D cell culture, tissue engineering

## Abstract

Mesothelial cells are specific epithelial cells that are lined in the serosal cavity and internal organs. Nonetheless, few studies have explored the possibility to culture mesothelial cells in a three-dimensional (3D) scaffold for tissue engineering applications. Towards this end, we fabricated macroporous scaffolds from gelatin and gelatin/hyaluronic acid (HA) by cryogelation, and elucidated the influence of HA on cryogel properties and the cellular phenotype of mesothelial cells cultured within the 3D scaffolds. The incorporation of HA was found not to significantly change the pore size, porosity, water uptake kinetics, and swelling ratios of the cryogel scaffolds, but led to a faster scaffold degradation in the collagenase solution. Adding 5% HA in the composite cryogels also decreased the ultimate compressive stress (strain) and toughness of the scaffold, but enhanced the elastic modulus. From the in vitro cell culture, rat mesothelial cells showed quantitative cell viability in gelatin (G) and gelatin/HA (GH) cryogels. Nonetheless, mesothelial cells cultured in GH cryogels showed a change in the cell morphology and cytoskeleton arrangement, reduced cell proliferation rate, and downregulation of the mesothelium specific maker gene expression. The production of key mesothelium proteins E-cadherin and calretinin were also reduced in the GH cryogels. Choosing the best G cryogels for in vivo studies, the cell/cryogel construct was used for the transplantation of allograft mesothelial cells for mesothelium reconstruction in rats. A mesothelium layer similar to the native mesothelium tissue could be obtained 21 days post-implantation, based on hematoxylin and eosin (H&E) and immunohistochemical staining.

## 1. Introduction

Mesothelial cells are specific epithelial cells that are lined in the serosal cavity (pleura, pericardium, and peritoneum) and internal organs. Their primary function is to provide a non-stick, frictionless protective barrier that facilitates the relative movement of tissues and organs within the serosal cavity [1]. The normal peritoneum is composed of a monolayer of mesothelial cells on the basement membrane as mesothelium, and is supported by a thin layer of connective tissue containing cells, blood vessels, and lymphatic vessels. Significantly increasing abnormalities in the mesothelium could be observed for patients on peritoneal dialysis with the length of time on dialysis, where the mesothelial cells are separated from the basement membrane, and in some cases, completely disappear [2]. As the peritoneum consists only of the connective tissue under mesothelial cells, the loss of peritoneal mesothelial cells is deemed to be associated with the development of ultrafiltration failure after long-term peritoneal dialysis [3].

In tissue engineering, cells can be seeded onto a three-dimensional (3D) artificial structure, called a scaffold. The scaffold provides mechanical support primarily in the formation of tissue engineered cell/scaffold constructs, which could be subsequently implanted into a host with defective tissues [4]. The biodegradable scaffold should have open interconnected macroporous network to allow for unimpeded cell penetration and the transport of oxygen, waste products, and nutrients. The 3D scaffold could also act as an artificial extracellular matrix (ECM), and serves as a template to guide cell adhesion, proliferation, and tissue development.

The cryogelation process could fabricate 3D scaffolds with a macroporous structure and allow for effective control over the pore size using ice crystals as templates [5]. During the cryogelation process, a polymer precursor solution was mixed with a crosslinking agent and allowed to undergo consecutive freezing, chemical crosslinking, and thawing steps [6]. As the polymer solution is reacted at a subzero temperature, most of the solvent freezes into ice crystals so as to concentrate the reactants; the cross-linking reaction is therefore carried out at a high solute concentration in order to form a dense network structure that enhances the mechanical strength of the cryogel scaffold [7,8]. Therefore, as cryogels can withstand high levels of deformation, including tensile, compressive, or flexural strains [9], it could be suggested as a suitable scaffold for a mesothelial cell culture and be used in the repair of the mesothelium layer in the peritoneum. Indeed, as cryogels are endowed with important properties, like a highly porous structure, pore interconnectivity, mechanical stability and flexibility, and good swelling in an aqueous solution, they are applied as scaffolds in many tissue engineering applications [10,11,12,13].

As one of the chief components of the basal lamina, collagen contributes to cell proliferation and migration; in addition, it can facilitate the integration of mesothelium. Compared with collagen, gelatin is a partially degraded product from collagen, with a lower antigenicity. Gelatin is not only more cost-effective than collagen as a scaffold material, but also is similar to collagen, which contains the important arginine–glycine–aspartic acid (RGD) amino acid sequence needed to enhance cell attachment [14,15]. Therefore, gelatin has been used alone, or by blending with other natural or synthetic biomaterials to produce different types of structures, including microparticles, nanoparticles, electrospun nanofibers, and in-situ gelling scaffolds for different tissue engineering applications [16].

Hyaluronic acid (HA) is a naturally polysaccharide composed of repeating disaccharide units of N-acetyl-D-glucosamine and D-glucuronic acid, linked by alternating (1→3) and (1→4) linkages. As one of the important ECM components, HA can increase cell attachment and cell migration into a tissue engineering scaffold, because of its intrinsic swelling and high water-retention properties. Thus, HA has been used alone, or cross-linked with other biological materials, for use in tissue engineering, such as skin, fat, bone, and cartilage [17].

In view of the successful use of gelatin and HA-based scaffolds in many tissue engineering applications, we hypothesized that cryogel scaffolds fabricated from gelatin and gelatin/HA, whose microenvironment mimics that of mesothelium ECM, would be suitable for a 3D culture of mesothelial cells. The purpose of this study is therefore to first fabricate macroporous, flexible cryogel scaffolds based on gelatin, and to elucidate the influence of HA on the physico-chemical properties of the cryogel scaffold, followed by studying the cellular response of seeded mesothelial cells. After identifying the best cryogel scaffold from in vitro experiments, mesothelium regeneration in vivo is followed by implanting a cell/scaffold construct into a rat abdomen for treating the peritoneum defect.

## 2. Results and Discussion

### 2.1. Synthesis and Characterization of Gelatin (G) and Gelatin/Hyaluronic Acid (HA) Cryogels

**Figure 1:**
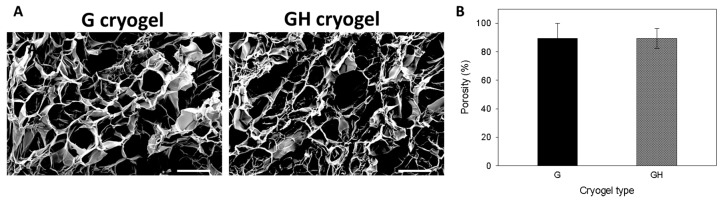
The SEM micrographs (**A**) and porosity (**B**) of gelatin (G) and gelatin/hyaluronic acid (GH) cryogels. Bar = 100 μm.

A macroporous structure of a cryogel scaffold is important in order to allow for cell penetration into the scaffold, and not to inhibit the cell growth and ECM secretion of the seeded cells. The morphology of the cryogel scaffolds was examined by scanning electron microscopy (SEM), where both gelatin (G) and gelatin/hyaluronic acid (GH) cryogels revealed a highly porous structure with an interconnected open pore morphology (Figure 1A). The average pore size from the SEM observations was estimated to be 88.3 ± 20.7 μm and 102.7 ± 39.9 for G and GH, respectively, with no significant difference found between them. The cryogel scaffolds also exhibited a similar porosity, close to ~90% (Figure 1B), which is considered to be beneficial for cell ingrowth and survival.

**Figure 2:**
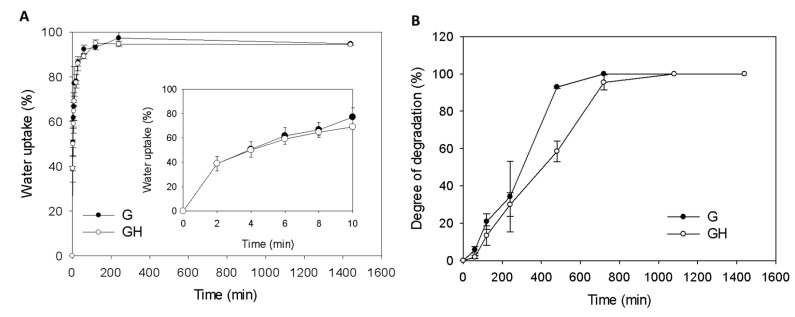
The water uptake kinetics in phosphate buffered saline (PBS) (**A**) and degradation kinetics in collagenase (**B**) of G and GH cryogels.

The water uptake kinetics and swelling ratio of the cryogel scaffolds was determined in phosphate buffered saline (PBS). In both cryogels, most of the water absorption occurred within the first 20 min, and reached a constant value after 200 min (Figure 2A). The swelling ratio calculated from the equilibrium water uptake was higher for G (18.2 ± 1.3) than GH (16.1± 1.9), but there was no significant difference between them. The water uptake kinetics was analyzed, and the results verify the satisfactory prediction of water diffusion into cryogels, with the fitted parameters’ (*k* and *n*) values being 28.49 and 0.42 for G (*r*^2^ = 0.998), respectively, and 30.66 and 0.36 for GH (*r*^2^ = 0.999), respectively. The mechanism of water diffusion within cryogel scaffolds could be inferred from the values of *n* for the disk-shaped cryogel samples, to be a Fickian type diffusion with *n* ≤ 0.5 [18].

The degradation studies showed ~30% degradation in collagenase at 37 °C in 4 h, and quantitative degradation after 20 h (Figure 2B). That degradation of G was faster than GH in a collagenase solution (Figure 2B).

**Figure 3:**
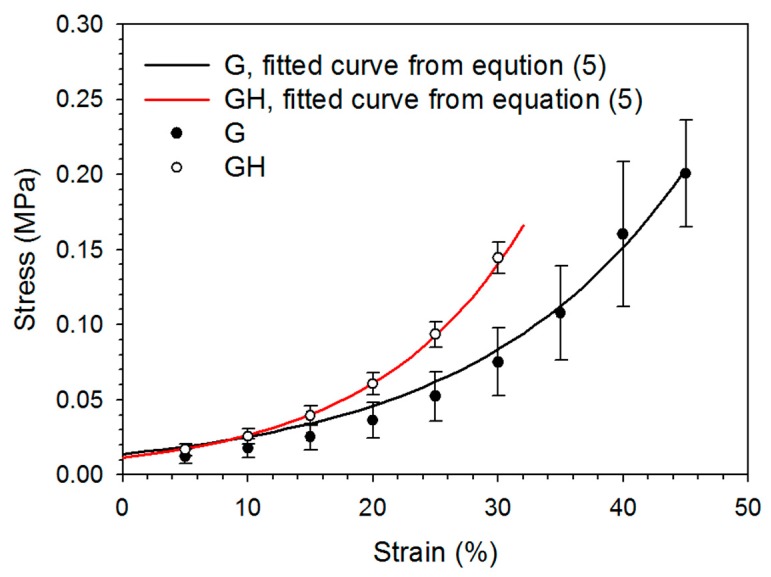
The typical compressive stress–stain curves of the G and GH cryogels. The lines are fitted curves from Equation (5).

The compressive stress–strain behavior of cryogels was non-linear, without an obvious linear elastic region (Figure 3). The incorporation of HA significantly increased the elastic modulus and stiffness up to the failure point, but decreased the toughness (Table 1). There is also a significant difference in the ultimate stress and ultimate strain, with G exhibiting a higher compressive strain and withstanding higher stress at failure point than GH (Table 1).

### 2.2. In Vitro Cell Culture

**Figure 4:**
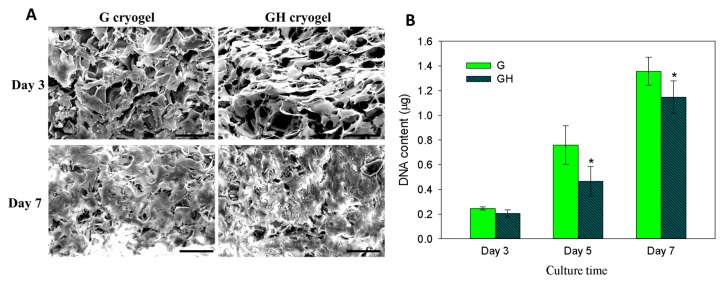
The cell morphology from SEM observation (**A**) and cell proliferation from DNA assays (**B**) of mesothelial cells cultured in G and GH cryogels. Bar = 50 μm. * *p* < 0.05 compared with G.

From the SEM observations of the cell-seeded cryogels, the mesothelial cells were mostly polygonal in shape, resembling a typical cobblestone pattern of mesothelial cells, on day three (Figure 4A). With the increase of culture time to seven days, the cells became more elongated, but the general phenotype remained. More cells, together with their secreted ECM, were also found to fill the pores within the cryogel scaffolds. Overall, the SEM images clearly supported the mesothelial characteristics of the seeded cells with a polygonal cell shape, with the microvilli visible on the surfaces of the cells. To further determine the cell proliferation, a cell number was compared between the G and GH cryogels, based on the DNA content per scaffold (Figure 4B). No significant difference in the DNA content was found on day three, and the mesothelial cells steadily proliferated up to day seven. Nonetheless, the cell number in G was significantly higher than that in GH on days five and seven, indicating that the incorporation of HA in the cryogel formulation may adversely affect cell proliferation.

**Figure 5:**
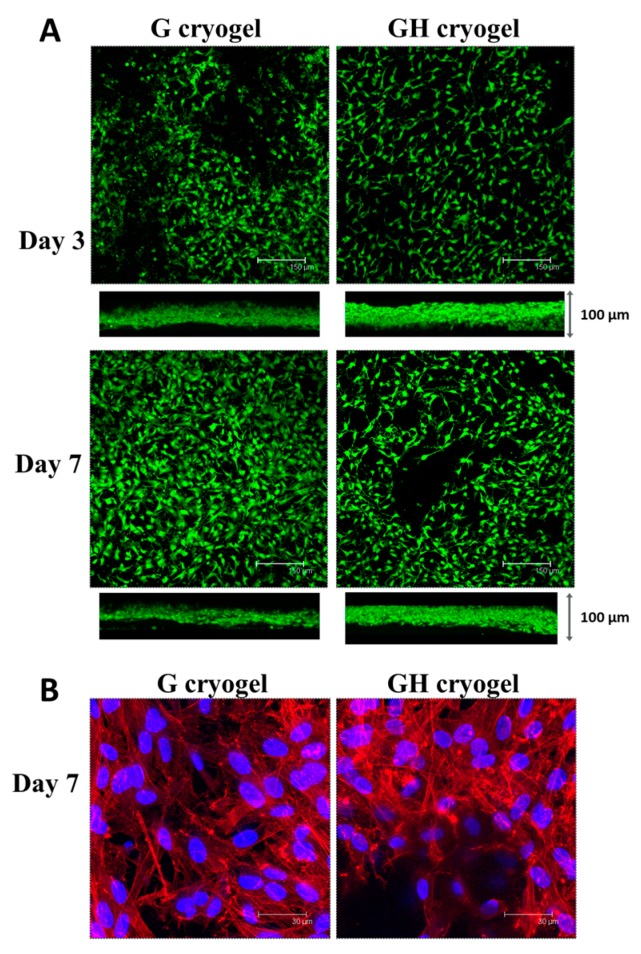
Confocal microscopy observation of mesothelial cells cultured in G and GH by live/dead (**A**) (bar = 150 μm) and nucleus/cytoskeleton staining (**B**) (bar = 30 μm). The live cells were stained green and the dead cells were stained red in (**A**), while the cell nuclei were stained blue by Hoechst 33342 and the actin cytoskeleton was stained red by rhodamine-phalloidin in (**B**). Both the merged top-view image and cross-sectional-view image are included in (**A**).

From the confocal microscopy analysis, the live/dead cell viability assays demonstrated a high cell viability in both cryogels, irrespective of culture time, with no dead cells (red) observed on days three and seven (Figure 5A). The top- and cross-section views indicated a good cell proliferation and penetration with a thick cell layer, increasing with the culture time, was found within the cryogel because of the macroporous nature of the scaffold. However, more live cells were observed on day seven in G, which is consistent with the DNA assays in Figure 4B. To reveal the cell morphology, the cell nucleus and cytoskeleton of the mesothelial cells cultured in the cryogels at the end of cultured period (seven days) were stained with rhodamine-phalloidin and Hoechst 33342, and were observed by confocal microscopy (Figure 5B). Although close to round shaped nuclei (blue) were observed for the mesothelial cells in both cryogels, there appeared to be a difference in the organization of the cellular cytoskeleton (red), with cells in the GH associated with more prominent, thick, actin-rich microfilaments that were arranged in stress fibers.

**Figure 6:**
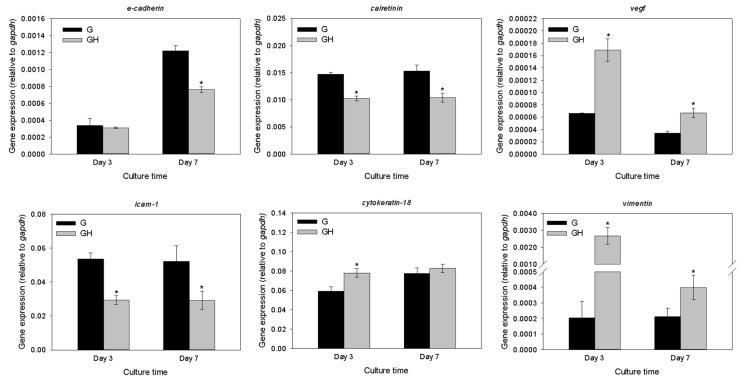
Gene expression of the mesothelial cells cultured in G and GH from a quantitative real-time polymerase chain reaction (qRT-PCR). * *p* < 0.05 compared with G.

The gene expression of the mesothelial cell markers (*E-cadherin*, intercellular adhesion molecule (*ICAM-1*), *calretinin*, *cytokeratin-18*, vascular endothelial factor (*VEGF*), and *vimentin*) were detected using a quantitative real-time polymerase chain reaction (qRT-PCR) to compare the relative mRNA levels for the cells cultured in G and GH at different times after normalizing with glyceraldehyde 3-phosphate dehydrogenase (GAPDH; Figure 6). The expression of the *E-cadherin*, *ICAM-1*, and *calretinin* genes were significantly upregulated for the cells cultured in G over GH, regardless of the culture period, except for *E-cadherin* on day three. On the contrary, the *VEGF* and *vimentin* genes were significantly downregulated in G compared to GH on day three and day seven. Although the mesothelial cells in G showed a similar *cytokeratin-18* gene expression as GH on day seven, the relative mRNA value was also significantly lower on day three.

**Figure 7:**
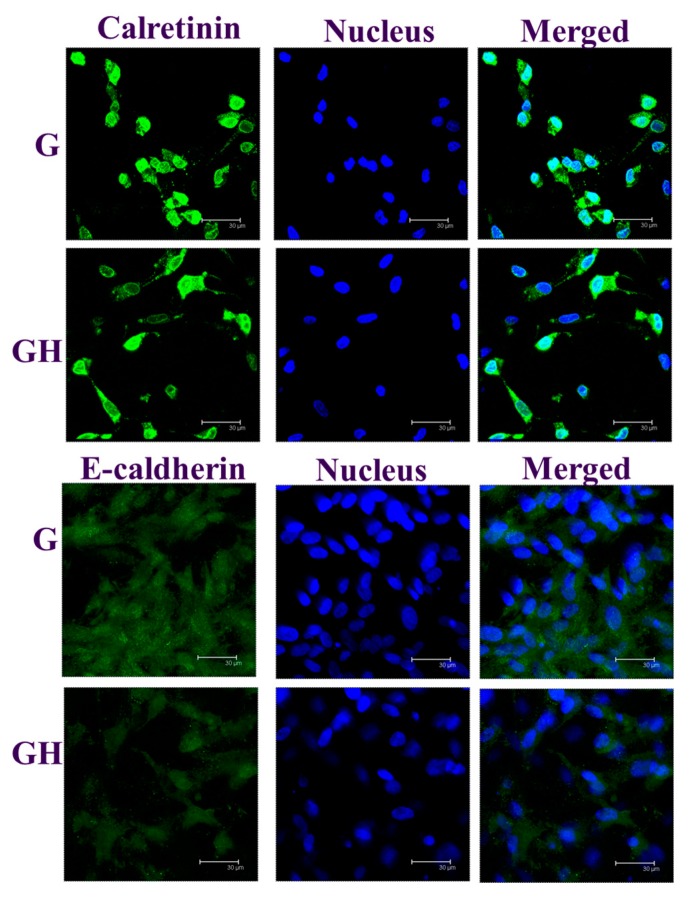
The immunofluorescence (IF) staining of calretinin and E-cadherin of the mesothelial cells cultured in G and GH for seven days. The protein was stained green by a fluorescein isothiocyanate (FITC)-conjugated secondary antibody, while the nuclei were stained blue by Hoechst 33342. Bar = 30 μm.

Immunofluorescence (IF) staining was used to reveal the production of the mesothelial marker proteins E-cadherin and calretinin on day seven, with cells identified from the nuclear staining (Figure 7). Consistent with the gene expression in Figure 6, the production of both E-cadherin and calretinin were more elevated in G than in GH. A semi-quantitative comparison of the protein production using PAX-it image analysis microscopy software (normalizing the area percentage of the green fluorescence with the number of nucleus in a single image) also indicated the dominance of G over GH in mesothelial marker protein production, as the value is 1.82 (G) and 1.58 (GH) for calretinin, and 1.30 (G) and 0.48 (GH) for E-cadherin.

### 2.3. In Vivo Studies

**Figure 8:**
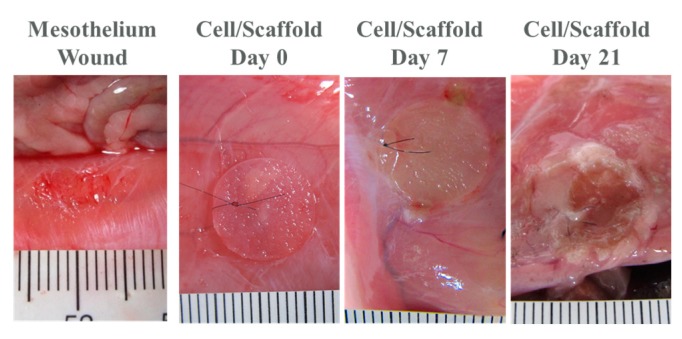
Gross view of the initial mesothelium wound and the transplanted cell/scaffold constructs at different time points post-implantation.

For the in vivo studies, rat parietal peritoneum was abraded to create a 10-mm diameter wound area, and was covered with a cell-scaffold construct (cultured in vitro for seven days beforehand) and examined 7- and 21-days post-implantation. During the course of the three-week in vivo animal experiment, we did not observe animal disability, infection, or death. One week after implantation, the cell/scaffold construct attached firmly to the defect (Figure 8). The scaffold was well recognized on the abdominal wall, and did not adhere to any internal organs. The formation of new tissues was scarce, and there was no ecchymosis around the scaffold, but local edema was noted. After three weeks, the construct became thinner around the edge because of biodegradation, but a thin layer of scaffold could still be identified attaching firmly to the abdominal wall, and free from adhesion with the surrounding tissues. The edges of the scaffold are smoother than before and edema is rare (Figure 8).

**Figure 9:**
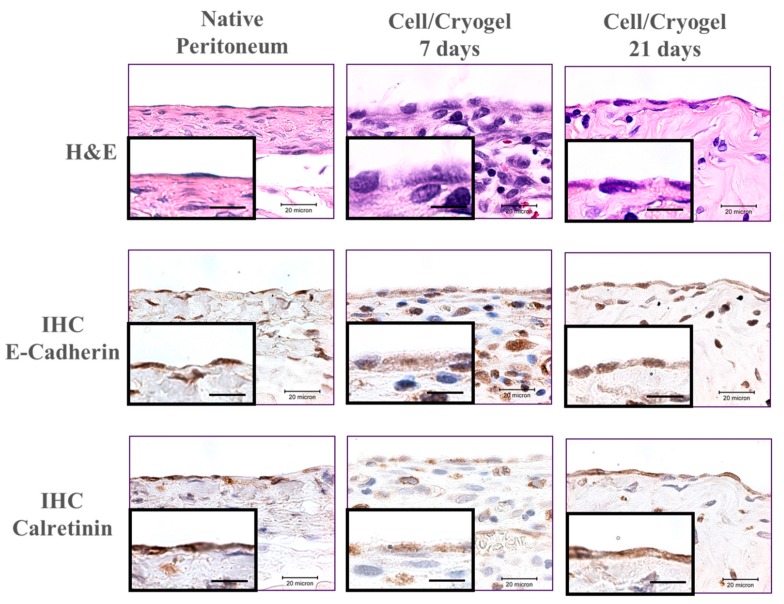
Hematoxylin and eosin (H&E) staining and immunohistochemical (IHC) staining of E-cadherin and calretinin of the cell/cryogel constructs 7- and 21-days post-implantation (bar = 20 μm). Native peritoneum tissue was used for comparison. The inserts are enlarged views on the surface of the specimen (bar = 10 μm).

From the histological examination, the hematoxylin and eosin (H&E) staining showed polygonal-shaped mesothelial cells in the cryogel scaffold on day seven, which showed a typical cobblestone pattern (Figure 9). After 21 days, mesothelial-like squamous cells were observed on the surface of the cell-laden cryogel scaffold, which is similar to native peritoneum. The immunohistochemical (IHC) staining of E-cadherin and calretinin indicated that the key mesothelial marker proteins of the transplanted cells could be identified from the brown stained color, with an increasing staining intensity with the transplantation time. This indicates that the transplanted allografts could continuously secrete mesothelial cell marker proteins. Overall, the transplanted cell/cryogel scaffold revealed a close similarity pattern of cell morphology and stained protein intensity to those of native peritoneum on day 21.

## 3. Discussion

In the study, we used cryogelation to fabricate a 3D macroporous scaffold for the culture of mesothelial cells. The peritoneal membrane is composed of a single layer of flattened mesothelial cells that have some characteristics of epithelial cells, attaching to the surface of a thin layer of collagenous tissue [19]. In the past, mesothelial cells were generally cultured in a two-dimensional (2D) fashion on tissue culture polystyrene (TCPS) or on dishes coated with gelatin or collagen [20]. For the transplantation of mesothelial cells, the mesothelial cells were seeded onto the upper surface of an artificial connective tissue sheet consisting of fibroblasts and collagen [21], or on fibrin gel matrix [22]. A transplantable peritoneal cell sheet was also reported using the cell sheet engineering approach with a temperature-responsive culture system, using fetal liver mesothelial cells [23], or an upper monolayer of mesothelial cells and underlying multilayered fibroblasts [24]. To the best of our knowledge, this study might be the first report of a 3D mesothelial cell culture in a cryogel scaffold for mesothelium tissue engineering.

For the preparation of the cryogel scaffolds, we used a non-toxic and biocompatible zero-length cross-linker, 1-ethyl-3-(3-dimethylaminopropyl) carbodiimide (EDC), to cross-link the adjacent gelatin/gelatin or gelatin/HA molecules, by forming covalent amide bonds [25]. During the fabrication of the GH cryogels, HA was first reacted with EDC to activate its carboxyl groups, and then the activated groups reacted with the primary amine group of the lysine residue in the gelatin. This method maximizes intermolecular cross-linking and minimizes intramolecular covalent bond formation within gelatin, and produces a robust cryogel with a high porosity.

Overall, gelatin and HA are good base materials in tissue engineering. The successful formation of intermolecular covalent linkages between gelatin–gelatin and gelatin–HA during cryogel fabrication could be justified from the macroporous structure after cryogelation at −17 °C, which promotes the formation of pores in micrometer scales from ice crystal growth within the gelling solution. The pore size may affect cell proliferation and growth. Previous studies have reported that a pore size ranging from 63–150 μm was beneficial for the cell culture [26,27], while a higher than 80% porosity is expected to be beneficial for cell ingrowth and survival [11]. Therefore, both of our scaffolds were endowed with a suitable pore size and porosity for the 3D mesothelial cell culture (Figure 1A,B).

The water binding ability is an important feature for a tissue engineering scaffold, as the swelling increases the pore size to maximize the surface area/volume ratio, which facilitates the cell infiltration into the scaffolds during a 3D cell culture [28]. From Figure 2A, both cryogels are associated with very fast water uptake kinetics and a high swelling capacity, where the equilibrium swelling ratio is approaching 20. This rapid swelling behavior is a characteristic observed in response to porous and hydrophilic materials [27]. Also, because of the high porosity (~90%) of the cryogel (Figure 1B), the swelling ratio did not change upon introducing the high-water absorbing HA in the cryogel. Judging from the Fickian diffusion mechanism of water during the cryogel swelling stage, no capillary effect was expected during the swelling step in the water, which could be correlated with the cryogel structure from the SEM observation (Figure 1A), where large pores free from long-aligned channels could be observed. This will allow for the unrestricted passage of water into the inner part of the cryogel during the swelling process.

The in vitro degradation of cryogels at 37 °C in collagenase solutions could reveal the cryogel stability under physiologically-relevant conditions, as collagenase could specifically imitate the enzymatic response of the cryogel in vivo. Collagenase induces the proteolytic cleavage at the peptide bond between glycine and a neutral amino acid (X) in the Pro–X–Gly¬–Pro amino acid sequence that occurs frequently in gelatin. The degradation studies using collagenase thus mimic the possible deterioration of the cryogels in vivo, and the endorsed biodegradable scaffold is suitable as a cell carrier for mesothelial cells (Figure 2B). That the degradation studies show a faster degradation rate of G than GH correlates well with the replacement of 5% gelatin with HA in GH cryogel, as collagenase will only hydrolyze peptide bonds in gelatin. We also checked the degradation of cryogels in PBS at 37 °C, where only 13% and 16% degradation rates were found for G and GH, respectively, after eight weeks. Therefore, we concluded that the cryogel scaffold should retain its structure during the in vitro cell culture, but it is biodegradable after implantation in order to fulfill the requirement of a tissue engineering scaffold. After cross-linking, the porosity, pore structure, and hydrophilic properties make both cryogels a suitable scaffold for the cell culture. The cryogels are also endowed with the proper mechanical properties for tissue engineering applications from compression testing (Figure 3). However, the G cryogel is more flexible and tough (Table 1). This feature is conducive to the environment of internal organ peristalsis when implanted in the abdominal cavity.

The mechanical testing of the mesothelium tissue is difficult, as it is a single layer of flattened cells, 2.5 to 3 μm in thickness, which is difficult to remove intactly from the bottom layer of the fibrous connective tissue. Considering the mechanical properties of peritoneum tissue, the maximum stress and strain were shown to be 0.37 MPa and 26%, respectively, from tensile tests [29]. Also, using the tensile tests, the ultimate stress of porcine peritoneum was reported to be from 0.525 to 0.579 MPa, and the modulus was from 0.09 to 1.01 MPa [30]. Using an atomic force microscope, the maximum Young’s modulus value of the normal mouse peritoneum was 35.88 kPa [31]. The mechanical strength tensile testing showed that the bovine peritoneum/fascia had a 31% ultimate strain and 60.6 kN/m stiffness [32]. Overall, the mechanical properties of the cryogels (shown in Table 1) compared favorably with those of peritoneum tissue, considering the ultimate stress (strain), Young’s modulus, and stiffness.

An analysis of the SEM images revealed that the mesothelial cells attached well to the walls of the inner pores within the scaffold, and produced abundant ECM during the 3D culture in cryogel (Figure 4A), while the live/dead staining also showed that the number of viable cells increased with time from both the top- and cross-sectional views (Figure 5A), indicating that the cryogel could maintain the cell viability and promote cell proliferation. In addition, the analysis showed that the cells proliferated along the 3D distribution of the pores. Mesothelial cells are peritoneal lining monolayer cells that have certain characteristics of epithelial cells and secrete various substances associated with peritoneal homeostasis [19]. Mesothelial cells are usually cultured on a 2D plane, but generally, solid organ cells are grown in a 3D manner. We can demonstrate that cryogel could be a suitable 3D scaffold for the attachment, proliferation, and maintenance of the phenotype of the mesothelial cells, with the configuration of cytoskeleton shown from rhodamine-phalloidin staining (Figure 5B).

Regardless of the species (human, rodent, rabbit, or horse) or anatomical origin, mesothelial cells constitute a uniform population that lines the internal organs and body walls in the peritoneum, pleura, and pericardium [33]. Although mesothelial cells are derived from mesoderm, they are very similar to simple epithelial cells; therefore, they express epithelial markers and can undergo an epithelial–mesenchymal transition (EMT), a transdifferentiation mechanism that induces the loss of their epithelial characteristics and the expression of the mesenchymal phenotype [34,35]. However, EMT in mesothelial cells is not just a pathological event. During development, mesothelial cells expressing membrane glycoprotein mesothelin (Msln) produce endothelial cells, fibroblasts, and smooth muscle in intra-abdominal organs, including in the lungs, liver, heart, and intestine [36,37,38]. This is consistent with previous studies, demonstrating that adipocytes and other mesenchymal lineages can be differentiated from mesothelial cells in a culture [39,40].

On the other hand, after reconstituting the intact mesothelium, these cells return to the epithelial-like phenotype [41,42]. From the cytoskeleton and live/dead staining, it can be seen that the performance of the mesothelial cells could be the same as that of the mesenchymal cells using a 3D culture in the cryogel scaffolds. When the mesothelial cells are removed and cultured, it will drive the performance of mesenchyme. The mesothelial cells have the potential to undergo 3D proliferation, and can be similar to the mesenchymal tissue proliferation. Therefore, the culture process will be different from that of normal epithelial cells, and there will still be ways to increase the growth in a 3D environment. The DNA content analysis also showed that the attached cell number in the scaffold increased with time. This means that the proliferation and growth of the mesothelial cells is feasible in cryogel. Concerning the cell proliferation rate, it was altered by the incorporation of HA into the cryogel from the DNA content comparison between G and GH (Figure 4B). One study indicated that soluble sodium hyaluronate could increase the proliferation rate of the attached human peritoneal mesothelial cells during the 2D cell culture [43]. The reduced cell proliferation could be associated with several mechanisms, including the ways HA interacts with mesothelial cells to affect its proliferation. HA might have effects on ECM synthesis or structuring [44]. HA might modulate the effects of the growth factors through regulating the surface receptor expression, or might modulate cell proliferation directly by a receptor-mediated pathway [45]. The presence of the CD44 receptor on the mesothelial cells also suggests that the binding of HA to the CD44 receptor may play a crucial role in the CD44-mediated cell–cell interactions, cell–matrix interactions, and in signal transduction [46].

The gene expressions of the mesothelial cells, including *E-cadherin*, *ICAM-1*, *calretinin cytokeratin-18*, *VEGF*, and *vimentin*, were detected using qRT-PCR (Figure 6). Mesothelial cells are unique in that they are derived from the mesoderm, and express mesenchymal intermediate filament vimentin and desmin, which also express cytokeratin [47]. Cytokeratins are keratin proteins found in the cytoplasmic cytoskeleton of epithelial tissues. They are an important component of the intermediate filaments, and help the cells resist mechanical stress [48]. The expression of these cytokeratins in epithelial cells is highly specific to a particular organ or tissue. Vimentin plays an important role in maintaining the cellular structure of the organelles in the cytoplasm. It interacts with other structural proteins, such as microtubules, to make cells stiff and sturdy [49]. Vimentin is attached either laterally or terminally to the nucleus, endoplasmic reticulum, and mitochondria [49]. As an organizer of many key proteins, vimentin may be involved in cell signaling, migration, and attachment [50]. Vimentin is commonly used as a marker for mesenchymally-derived cells or cells undergoing EMT during normal development [51]. Mesothelial cells normally have a high keratin and a low vimentin content in vivo [52]. The *cytokeratin* expression was diminished and associated with an increment in the *vimentin* expression [53]. Many transforming epithelial cells also change their intermediate filaments from cytokeratin to vimentin, and the cytoskeletal transformation seems to be necessary for the beginning of the transformation process [54]. Comparing the gene expression patterns of *cytokeratin* and *vimentin*, *cytokeratin* was significantly more prominent than *vimentin* (Figure 6). This means that the characteristics of the epithelial cells continue to persist during cell division and proliferation during the 3D culture in cryogels. Nonetheless, the expression of *vimentin* was significantly up-regulated by the incorporation of HA in the cryogel, while the *cytokeratin* expression was largely maintained, which indicates that the G cryogel excels over GH in maintaining the phenotype of the seeded mesothelial cells (Figure 6).

Calretinin is a calcium-binding protein involved in calcium signaling, and is expressed in the mesothelial cells, in addition to certain neural tissues [55,56]. The exact function of calretinin is not well-defined, but it is thought to play a role in the cell cycle. Several immunohistochemical studies have suggested that it is a very useful marker for cells of the mesothelial lineage [57,58]. E-Cadherin is a calcium-dependent transmembrane epithelial protein that promotes intercellular adhesion [59]. The intercellular adhesion molecule E-cadherin appears to have a central role in the control of the epithelial-to-mesenchymal transition, as the loss of E-cadherin expression or function correlates with the ability of the epithelial cells to adopt a mesenchymal migratory and invasive phenotype [60]. Intercellular adhesion molecule-1 (ICAM-1, also called CD54) is localized to the plasma membrane and cytosol. Mesothelial cells characteristically express the adhesion molecule *ICAM-1*, whose expression is constitutive [61]. In addition, *ICAM-1* appears to be a potential marker that discriminates between mesothelial cells and fibroblasts [53]. The culture of mesothelial cells in the GH cryogel scaffold rendered the down-regulation of the mesothelium marker genes (*calretinin*, *E-cadherin*, and *ICAM1*; Figure 6). This underlines the use of G cryogels for the 3D culture of mesothelial cells in the persistence of epithelial cell characteristics during cell division and proliferation. Vascular endothelial growth factor (VEGF) is a potent proangiogenic factor involved in endothelial cell proliferation and vascular permeability [62]. However, VEGF is considered a main autocrine growth factor for mesothelial cells; an increase in its production might stimulate mesothelial cell growth [63]. It is reported that the mechanism underlying *VEGF* up-regulation in mesothelial cells is the EMT of these cells [64]. Furthermore, the local production of VEGF by transitional mesothelial cells appears to play an important role in the process leading to peritoneal angiogenesis [65]. The gene expression of *VEGF* was up-regulated in the presence of HA in the cryogel (Figure 6), indicating the maintenance of mesothelial cell characteristics in cryogels.

To confirm the gene expression patterns, the production of calretinin and E-cadherin was evaluated by IF staining, which could show the distribution and production of the proteins by seeded mesothelial cells within the cryogel scaffold (Figure 7). Specifically, abundant mesothelial-specific proteins could be identified to be lined along the cryogel pore wall, where mesothelial cells could be also identified from the nuclear staining. Consistent with the gene expression, the dominance of G over GH in the production of both proteins was confirmed from a semi-quantitative analysis with the normalized area percentage of the green fluorescence.

It has been reported that increased hyaluronan levels can induce EMT in mesothelial cells, which is essential for cell migration during wound healing and remesothelization [66]. HA fragments also possess proinflammatory properties that can activate the inflammatory cascade [67]. Therefore, mesothelial cells cultured in GH have a tendency to induce EMT, where the cell proliferation, protein expression, and gene expression were inferior to those in G. Based on studies where the G scaffolds are superior to the GH scaffolds for in vitro mesothelial cell cultures, we chose a G cryogel scaffold for the animal studies, and implanted a seven-day in vitro cultured cells/scaffold construct into the abdomen of a rat that was induced with a defective mesothelium. The scaffold retained its structure during the in vitro culture, but gradually degraded after 21 days post-implantation, with a discernible scaffold residue (Figure 8).

The mesothelial cells form a monolayer, known as the mesothelium, which lines the pleural, peritoneal, and pericardial cavities, with visceral and parietal surfaces covering the internal organs and body wall, respectively [33]. From the H&E staining, many inflammatory cells were found to infiltrate the scaffold, and were found within the scaffold on day seven because of the initial inflammatory response induced by foreign body reactions, which drastically decreased after 21 days (Figure 9). Most importantly, the uppermost layer of the cell/cryogel construct showed a mesothelial cell layer that closely resembles that in the native mesothelium from the close-up views in the inserts of each group on day 21. The IHC staining also endorsed the formation of a mesothelium layer similar to that of the native mesothelium on the top surface of the cell-scaffold construct, forming from the transplanted mesothelial cells that are associated with abundant key marker protein (calretinin and E-cadherin) secretion. Indeed, many inflammatory cells were noted on day seven, which disappeared on day 21 after the wounds healed. The uppermost layer of the scaffold was finally only covered with a monolayer of mesothelial cells when the scaffold was degrading. After 21 days post-implantation, the normal physiological manifestation of mesothelium was that the mesothelial cells at the visceral and parietal contact surfaces were monolayer distributed. As shown in Figure 9, the uppermost layer of the cell/scaffold is equivalent to the parietal peritoneum surface, and the mesothelial cells should not be distributed elsewhere, because they will not perform their normal physiological functions. It is expected that the new mesothelium will be remodelled, and a functional mesothelium developing from the allograft mesothelial cells will attach to the wound to repair the damaged mesothelium when the cryogel scaffold is completely degraded over times.

## 4. Materials and Methods

### 4.1. Materials

The ethyl-3-(3-dimethylaminopropyl) carbodiimide (EDC) was obtained from Acros Organics (Geel, Belgium). The gelatin (type A from porcine skin, 300 bloom) was purchased from Sigma-Aldrich (St Louis, MO, USA) and hyaluronic acid (sodium salt, average molecular weight = 1.3 × 10^6^ Da) were acquired from Bloomage Biotechnology (Jinan, China). HyClone Dulbecco’s Modified Eagles Medium (DMEM) and fetal bovine serum (FBS) were used for the cell culture and were purchased from Thermo Fisher Scientific (Waltham, MA, USA). The rhodamine-phalloidin (tetramethylrhodamine B isothiocyanate-phalloidin, TRITC-phalloidin) and Hoechst 33342 for the actin cytoskeleton and nucleus staining were purchased from Life Technologies (Carlsbad, CA, USA).

### 4.2. Preparation of Gelatin (G) and Gelatin-Hyaluronic Acid (GH) Scaffold by Cryogelation

Cryogels containing 5% gelatin and 5% gelatin/0.25% HA were prepared. The gelatin (10% *w*/*v*) and HA (0.5% *w*/*v*) solutions were prepared separately in a 0.1 M 2-(N-morpholino) ethanesulfonic acid (MES) buffer (pH 6.0). The gelatin solution was gently shaken in a 70 °C water bath until the gelatin flake was completely dissolved and cooled to room temperature. For the preparation of the gelatin (G) cryogel, 1 mL of a 10% gelatin solution was mixed with 1 mL of 4% 1-ethyl-3-(3-dimethylaminopropyl) carbodiimide (EDC) in a 0.1 M MES buffer (pH 6.0), in a 3 mL polypropylene syringe (inside diameter = 8 mm). The mixture was stirred at a low speed for 5 s, and placed in a water bath filled with −17 °C ethanol. The whole mixing and transfer step was completed within 1 min. The syringe mold was incubated in the bath for 16 h so as to complete the cross-linking reaction, and was then completely thawed at room temperature. For the gelatin/hyaluronic acid (GH) cryogel, the EDC powder was dissolved in a 1 mL 0.5% *w*/*v* HA solution prepared in a MES buffer (pH 6.0) to a final concentration of 4%. The solution was mixed for 30 min at room temperature so as to activate the carboxyl groups of HA. The HA solution (1 mL) was then mixed with an equal volume of 10% *w*/*v* gelatin solution prepared in a MES buffer (pH 6) and placed in a 3 mL polypropylene syringe (inside diameter = 8 mm) syringe mold. The same cryogelation procedure was followed as for the G cryogel. After receding from the syringe, the cryogel was cut into disc-shaped scaffolds (1 mm thickness × 8 mm diameter) using a surgical blade, and was thoroughly washed with deionized water at room temperature in order to remove any residual reactants. The cryogel scaffolds were dehydrated in gradient alcohol, followed by critical point drying for storage. For the in vivo studies, a 5 mL polypropylene syringe (inside diameter = 12 mm) was used to prepare the disc-shaped scaffolds (1 mm thickness × 12 mm diameter) for implantation, following the same preparation condition described before.

### 4.3. Characteristics of Cryogel

The microstructure of the scaffold of cryogel samples was examined with an S-3000N scanning electron microscope (SEM) from Hitachi (Tokyo, Japan), after gold sputter coating. The porosity of the cryogel was calculated using the ethanol displacement method [68]. To study in vitro degradation of cryogel, a collagenase (30 units/mL) solution was prepared in PBS (pH 7.4). The dry weight (*W*_1_) of the oven-dried cryogel samples were measured and placed in wells of a 24-well culture plate. Then, 2 mL of a collagenase solution was added to each well, and they were incubated at 37 °C. The cryogel samples were retrieved at different time points from the well, were rinsed with distilled deionized water, and dried overnight in an oven at 70 °C to a constant weight (*W*_2_). The degree of degradation was calculated from the following equation.

(1) $$\mathrm{Degree}\;\mathrm{of}\;\mathrm{degradation}\;\left(\%\right)=\frac{\left(W_{1}-W_{2}\right)}{W_{1}}\times 100$$

The water uptake and swelling of the cryogel scaffolds were studied in PBS (pH 7.4) following the gravimetric procedure. The cryogel samples were dried for 24 h at 70 °C and were weighed (*W_d_*) [69]. The dried samples were immersed in PBS at room temperature, followed by retrieving the wet samples at different time points. The retrieved samples were gently shaken and blotted with tissue paper in order to remove any excess water from the surface, and the weight of the swollen gel samples (*W_t_*) were determined immediately. The equilibrium weight (*W_eq_*) of the swollen sample was obtained after being immersed in PBS for 24 h at room temperature, where no measurable weight increase could be observed. To determine the swelling kinetics, the water uptake was calculated at different time points using Equation (2).

(2) $$\mathrm{Water}\;\mathrm{uptake}\;\left(\%\right)=\frac{W_{t}-W_{d}}{W_{eq}}\;\times 100$$

To investigate the diffusion of water in the cryogels, the swelling kinetics were fitted to Equation (3) [18].

(3) $$\frac{W_{t}-W_{d}}{W_{eq}-W_{d}}=kt^{n}$$

where *k* is a characteristic constant of the gel, *t* is time, and *n* is a characteristic exponent of the transport mode of water.

The swelling ratio was calculated from Equation (4).

(4) $$\mathrm{Swelling}\;\mathrm{ratio}=\;\frac{W_{t}-W_{d}}{W_{d}}$$

To determine the mechanical properties of the cryogels, unconfined quasi-static compression tests using wet cryogel samples were performed. The samples were soaked in PBS for 24 h prior to testing, and the testing was carried out at 37 °C using an ElectroForce 5200 BioDynamic Test Instrument from TA Instruments (New Castle, DE, USA). A 250 N load cell was used to apply the compression load at a 0.05 mm/s crosshead speed. Based on the stress (σ) vs strain (ε) curve of the load–deformation data subject to the uniaxial stress, the ultimate stress and ultimate strain at the failure point and compressive Young’s (elastic) modulus were determined. To model the non-linear compressive behavior of a cryogel sample, Equation (5) was used to fit the σ−ε curve, up to failure [70].

(5) $$\sigma =Ae^{\left(B\varepsilon -1\right)}$$

where A and B are the fitted empirical parameters. The tangential Young’s moduli (slope of the tangent to the σ−ε curve) at 10%, 20%, and 30% strains were calculated using Equation (5), while the toughness (compressive strain energy to failure) representing the energy required to deform the sample was obtained from the region under the curve.

### 4.4. Culture of Mesothelial Cells in Cryogel Scaffolds

#### 4.4.1. Isolation and Harvest of Mesothelial Cells

To harvest the mesothelial cells from the lower abdomen of a Sprague-Dawley (SD) rat, the abdomen wall was pre-sterilized with a beta-iodine solution. The skin was picked up with forceps on the abdomen wall, and was gently cut with a blade to separate the it from the peritoneum. The procedure was approved by the Institutional Animal Care and Use Committee of Chang Gung University (IACUC approval no. CGU106-045, approved on 6/9/2017), and followed the standards of the Association for Assessment and Accreditation of Laboratory Animal Care. The peritoneum was diced into pieces of a 2 × 2 cm^2^ area using scissors, in a sterile procedure. The diced peritoneum sample was washed extensively with equal volumes of PBS, and the ECM was digested with 0.2% collagenase at 37 °C, and was shaken at 20 rpm for 30 min. The peritoneum was discarded and the liquid was filtered through a 70 μm pore-size filter to remove the debris. The enzyme activity was neutralized by adding 20 mL of a culture medium consisting of DMEM with 10% FBS, and centrifuged at 100× *g* for 5 min in order to obtain a high-density cell pellet. The supernatant was discarded, and the cell pellet was re-suspended in a culture medium and incubated at room temperature.

#### 4.4.2. In Vitro Cell Culture

Disc-shaped cryogel scaffolds (1 mm thickness × 8 mm diameter) were sterilized with 75% ethanol for 24 h, and rinsed two times with PBS before being placed in 24-well culture plates for cell seeding. An aliquot of a 10 μL cell suspension (10^7^ cells/mL) was loaded onto the surface of the cryogel scaffold. The cell-seeded cryogels were incubated at 37 °C for 2 h so as to allow for cell adhesion, and were transferred to a new well, followed by adding 1 mL of a culture medium to each well. The cells were cultured at 37 °C in 5% CO_2_, with a medium change every three days.

#### 4.4.3. SEM Analysis

After three and seven days of the cell culture, the mesothelial cell/cryogel constructs were examined by SEM. The constructs were washed with PBS and the cells in the scaffold were fixed using 2.5% glutaraldehyde at room temperature for 3 h. All of the samples were washed another three times with PBS, and were treated with increasing concentrations of ethanol from 50% to 100%, and were dried in a critical point dryer. The completely dried samples were sputter coated with gold and mounted on a S-3000N SEM (Hitachi, Tokyo, Japan) in order to observe the cell morphology.

#### 4.4.4. DNA Quantification

The cell-seeded scaffolds were harvested at predetermined times, and were digested for 24 h in papain solutions (55 mM sodium citrate, 150 mM sodium chloride, 5 mM cysteine hydrochloride, 5 mM ethylenediaminetetraacetic acid (EDTA), and 0.2 mg/mL papain) at 60 °C to determine the DNA content. The DNA content was determined with Hoechst 33258 in an enzyme-linked immunosorbent assay (ELISA) reader (excitation = 360 nm; emission = 460 nm) [71].

#### 4.4.5. Live/Dead Staining

The live/dead viability/cytotoxicity kit was purchased from Molecular Probes Inc. (Eugene, OR, USA) and was used to qualitatively evaluate the cell viability. After being cultured for seven days in vitro, the cell-seeded scaffolds were washed with PBS and stained with 1 mL of a staining solution. The staining solution was prepared by diluting 3 μL of calcein AM and 5 μL of ethidium homodimer-1 (EthD-1) reagents in 10 mL of PBS at 37 °C for 15 min. The live and dead cells were stained green and red separately, with calcein AM and EthD-1, which could be imaged under a confocal laser scanning microscope (Leica TCS SP2, Leica Microsystems, Wetzlar, Germany) at an excitation/emission wavelength of 494/517 nm and 528/617 nm.

#### 4.4.6. Cell Cytoskeleton Staining

To assess the cytoskeletal structure of the mesothelial cells within the cryogels, the cell/scaffold constructs after the seven-day culture were fixed in 10% *w*/*v* formaldehyde in PBS for 30 min at room temperature. The sample was washed with PBS three times, and treated with 0.1% Triton X-100 in PBS for 10 min for the permeabilization of the cell membrane. The constructs were then immersed in 1 μg/mL rhodamine-phalloidin for 30 min in the dark, and were washed three times with PBS. The cell nuclei were counterstained with 10 μg/mL Hoechst 33342 in PBS for 30 min, and were immediately visualized for their cytoskeletal arrangements using a Leica TCS SP2 confocal laser scanning microscope. The F-actin cytoskeleton emits a red fluorescence and the nucleus is stained blue using an excitation/emission wavelength of 540/570 nm for the rhodamine-phalloidin and 350/461 nm for the Hoechst 33342.

#### 4.4.7. Quantitative Real-Time Polymerase Chain Reaction (qPCR)

The expression of the mesothelial marker genes was analyzed by a quantitative real-time polymerase chain reaction (qRT-PCR) using standard protocols of cDNA synthesis and RNA isolation. The total RNA from each sample was isolated using the TRIzol reagent, according to the standard protocol. The isolated RNA was dissolved in diethylpyrocarbonate (DEPC)-treated water, and the amount of RNA was determined by measuring the absorbance at 260 nm (OD_260_) with a NanoDrop 2000 spectrophotometer (Thermo Fisher Scientific, Waltham, MA, USA). The RNA quality was verified from OD_260_/OD_280_ measurements. The cDNA was synthesized using a Maxime RT PreMix Kit, according to the standard procedures. Glyceraldehyde-3-phosphate dehydrogenase (*GAPDH*) acted as a housekeeping control. Amplification was conducted for 45 cycles in a PCR thermo cycler. Each cycle consisted of 10 min at 95 °C for denaturation; 30 s at 95 °C, as obtained from the melting curves for annealing; and 1 min at 60.9 °C (*calretinin*) or 67.1 °C (*cytokeratin-18*, *E-cadherin*, *GAPDH*, *ICAM-1*, *VEGF*, and *vimentin*) for extension. A MiniOpticon real-time PCR system (Bio-Rad CFD-3120, Bio-Rad, Hercules, CA, USA) was used for the qPCR measurements using the SYBR Green qPCR Supermixes. The primer sequences were *E-cadherin* (forward: 5’ AAGGGCTTGGATTTTGAGG 3’; reverse: 5’ AGATGGGGGCTTCATTCAC 3’), *ICAM-1* (forward: 5’ GCCTGGGGTTGGAGACTAAC 3’; reverse: 5’ CTGTCTTCCCCAATGTCGCT 3’), *vimentin* (forward: 5’ TGCCAACCGGAACAACGAT 3’; reverse: 5’ AATTCTCTTCCATTTCACGCATC 3’), *cytokeratin-18* (forward: 5’ CAGATACAGGGTGCAGATGGAG 3’; reverse: 5’ GGGCGTCGTTGAGACTGAAATC 3’), *calretinin* (forward: 5’ TATCCAGCAGCTCACCACCTAC 3’; reverse: 5’ GAGAGGTCTGGGAAGGAGTTTC 3’), *VEGF* (forward: 5’ TGAGACCCTGGTGGACATCT 3’; reverse: 5’ CTCCTATGTGCTGGCTTTGG 3’), and *GAPDH* (forward: 5’ CACCATCTTCCAGGAGCGAG 3’; reverse: 5’ GGCGGAGATGATGACCCTTT 3’).

#### 4.4.8. Immunofluorescence (IF) Staining

For the immunofluorescence (IF) staining of E-cadherin and calretinin, the cell/cryogel constructs that were cultured for seven days were fixed with 10% *w*/*v* formaldehyde at 4 °C for 1 h. After fixation, the constructs were washed three times in PBS containing 0.1% Tween 20 (PBST) for 10 min. The non-specific binding sites were blocked with a Hyblock blocking buffer for 1 min, and washed three times in PBST for 30 min. An anti-calretinin (rabbit polyclonal, Thermo Fisher Scientific PA5-16681, 1:400 in PBST) or E-cadherin (rabbit polyclonal, Thermo Fisher Scientific PA5-32178, 1:500 in PBST) primary antibody was added and incubated overnight at 4 °C. The samples were washed three times in PBST for 10 min, and then incubated in a fluorescein isothiocyanate (FITC) AffiniPure goat anti-rabbit IgG (H+L) secondary antibody (Jackson Immuno Research Laboratories Inc., 111-095-003) for 1 h at 37 °C. After washing three times in PBST for 10 min, the construct was incubated for an additional 30 min at room temperature in 100 µg/mL Hoechst 33342 for nuclear staining, and washed three times in PBST for 15 min. The sample was observed by a confocal laser scanning microscope (Leica TCS SP8, Leica Microsystems, Wetzlar, Germany) at excitation/emission wavelengths of 490/525 nm for FITC, and 350/461 nm for Hoechst 33342. The PAX-it!^TM^ image analysis software (version 7.8.1, MIS Inc., Villa Park, IL, USA) was used for the semi-quantitative evaluation of the E-cadherin and calretinin produced by the mesothelial cells.

### 4.5. In Vivo Studies

The animal protocols were approved by the Institutional Animal Care and Use Committee of Chang Gung University (IACUC approval no. CGU106-045, approved on 6/9/2017). The male SD rats weighed between 300 to 380 g were used for the in vivo experiments. The rats underwent surgery during an inhalation induction of isoflurane anesthesia, then depilating the lower abdomen area. After the abdomens were cleaned with an alcohol and betadine solution, a C-shaped incision with a 5-cm diameter was made using sterile techniques for laparotomy. The parietal peritoneum was abraded with a sterile tooth brush for 100 strokes in order to create a 10-mm diameter wound area. After the abraded surface formation, the wound was covered with a cryogel (12 mm diameter × 1 mm thickness) seeded with 3 × 10^5^ mesothelial cells, which was cultured for seven days in vitro before implantation. The abdominal wall was closed using 3–0 nylon running suture, without creating any trauma on abdominal wall, and the skin incision was closed with a 3–0 nylon interrupted suture. The animals were sacrificed by CO_2_ inhalation 7- and 21-days post-implantation. The abdominal cavities were opened in a U-shaped incision, and were examined from gross view observation. For the histological examination, the specimens were collected and fixed in a 10% buffered formaldehyde solution for 48 h, and were embedded into paraffin. The paraffin sections were cut at 5 μm and were subject to hematoxylin and eosin (H&E) stain for the histological evaluation. For the immunohistochemical (IHC) staining, the sections were deparaffinized and rehydrated. The sections were then washed three times in PBST for 5 min. The non-specific binding sites were blocked with hydrogen peroxide for 10 min, and the sections were washed three times in PBST for 5 min again. The sections were then incubated for an additional 60 min at room temperature in a rabbit anti-calretinin primary antibody (rabbit polyclonal, Thermo Fisher Scientific PA5-16681, 1:600 in PBST) or rabbit anti-E-cadherin primary antibody (rabbit polyclonal, Thermo Fisher Scientific PA5-32178, 1:800 in PBST), in a humid environment. The slides were washed in PBST for 5 min and were treated with an HRP Polymer Quanto (Thermo Fisher Scientific, Waltham, MA, USA) for 10 min, followed by a washing step for 5 min. The peroxidase activity was visualized using 3-diaminobenzidine (DAB) as the substrate, by incubation for 1 min. The sections were finally counterstained with hematoxylin for 30 s, and were observed under an inverted optical microscope.

### 4.6. Statistical Analysis

All of the quantitative data were expressed as mean ± standard deviation (SD), and the statistical analysis was performed by a one-way analysis of variance (ANOVA) least significant difference (LSD) test in order to determine the significant difference (*p* < 0.05).

## 5. Conclusions

This study demonstrates the feasibly of a cryogel scaffold for a 3D culture of mesothelial cells. The incorporation of HA in gelatin-based cryogel was found to modulate the scaffold properties, in addition to influencing the cellular response of the seeded mesothelial cells. Although there was no change in the pore structure and water absorbing properties, GH showed a faster degradation in the collagenase solution and a higher elastic modulus than G cryogel. From the in vitro cell culture, the rat mesothelial cells proliferated well in the cryogel scaffolds, although the cells cultured in GH showed a change in the cell morphology, cytoskeleton arrangement, and proliferation rate. The mesothelial cells in the cryogels also have a good phenotypic expression from qRT-PCR, as well as IF staining results. Nonetheless, the downregulation of the mesothelium specific maker gene, together with the reduced production of the key mesothelium proteins, E-cadherin and calretinin, was noted for the GH compared with G cryogel. Taken together, the HA incorporation in the cryogel may have an adverse effect on the mesothelial cell behavior during the 3D in vitro cell culture, pointing out the choice of G cryogel as the scaffold for tissue engineering applications, where in vitro cultured cell/scaffold constructs were used for neo-mesothelium formation in vivo. In animal studies, the G cryogel could be shown to provide a good vehicle for delivering mesothelial cells from a rat mesothelium wound model. After implantation, the scaffold degraded gradually in concomitant with the remodeling of the mesothelial cells into a parietal mesothelium, similar to the native mesothelial from H&E and IHC staining. It is expected that the damaged mesothelium could be repaired after the cryogel is completely degraded and the neo-mesothelium tissue attaches to the wound.

## Figures and Tables

**Table 1:** Mechanical properties of G and GH cryogels. Values are the mean ± standard deviation (SD) of five independent measurements.

Mechanical Property	G	GH
Compressive elastic modulus at 10% strain (MPa)	0.15 ± 0.05	0.22 ± 0.03 *
Compressive elastic modulus at 20% strain (MPa)	0.31 ± 0.11	0.52 ± 0.04 *
Compressive elastic modulus at 30% strain (MPa)	0.64 ± 0.23	1.26 ± 0.16 *
Compressive strain to failure (%)	45.0 ± 4.9	32.8 ± 2.2 *
Compressive stress to failure (MPa)	0.21 ± 0.04	0.17 ± 0.02 *
Toughness (kJ/m^3^)	35.0 ± 2.6	22.5 ± 5.4 *
Compressive stiffness at 0.2 mm displacement (kN/m)	4.45 ± 1.55	6.51 ± 0.67 *
Compressive stiffness at 0.4 mm displacement (kN/m)	8.99 ± 3.08	14.54 ± 0.84 *
Compressive stiffness at 0.6 mm displacement (kN/m)	18.20 ± 6.19	32.63 ± 3.68 *

* *p* < 0.05 compared with G.
